# Cross-talk between *Helicobacter pylori* and gastric cancer: a scientometric analysis

**DOI:** 10.3389/fcimb.2024.1353094

**Published:** 2024-01-31

**Authors:** Shanshan Yang, Shaodong Hao, Hui Ye, Xuezhi Zhang

**Affiliations:** ^1^ Department of Integrated Traditional Chinese and Western Medicine, Peking University First Hospital, Beijing, China; ^2^ Spleen-Stomach Department, Fangshan Hospital, Beijing University of Chinese Medicine, Beijing, China

**Keywords:** *Helicobacter pylori*, gastric cancer, hotspots and trends, high-cited papers, bibliometrics

## Abstract

**Background:**

*Helicobacter pylori* (HP) is considered a leading risk factor for gastric cancer (GC). The aim of this article is to conduct bibliometric and visual analysis to assess scientific output, identify highly cited papers, summarize current knowledge, and explore recent hotspots and trends in HP/GC research.

**Methods:**

A bibliographic search was conducted on October 24, 2023, to retrieve relevant studies on HP/GC research between 2003 and 2022. The search terms were attached to HP and GC. The main data were from the Web of Science Core Collection (WoSCC). Data visualization was performed using Biblioshiny, VOSviewer, and Microsoft Excel.

**Results:**

In HP/GC research, 1970 papers were retrieved. The total number of papers (Np) in HP/GC was growing from 2003 to 2022. China and Japan were in the leading position and made the most contributions to HP/GC. *Vanderbilt University* and the *US Department of Veterans Affairs* had the highest Np. The most productive authors were Peek Jr Richard M. and Piazuelo M Blanca. *Helicobacter* received the most Np, while *Gastroenterology* had the most total citations (TC). High-cited publications and keyword clustering were used to identify the current status and trends in HP/GC research, while historical citation analysis provided insight into the evolution of HP/GC research. The hot topics included the effect of HP on gastric tumorigenesis and progression, the pathogenesis of HP-induced GC (HP factors), and the mechanisms by which HP affects GC (host factors). Research in the coming years could focus on topics such as autophagy, gut microbiota, immunotherapy, exosomes, epithelial-mesenchymal transition (EMT), and gamma-glutamyl transpeptidase (GGT).

**Conclusion:**

This study evaluated the global scientific output in HP/GC research and its quantitative characteristics, identified the essential works, and collected information on the current status, main focuses and emerging trends in HP/GC research to provide academics with guidance for future paths.

## 1 Introduction

Gastric cancer (GC) remains a global public health concern (Sung et al., 2021), with the fifth most common cancer and the fourth leading cause of cancer-related death worldwide (Thrift et al., 2023). *Helicobacter pylori* (HP) infection is the main pathogenic factor for GC, which plays an essential regulatory role in GC incidence, development, and treatment. Approximately 4.4 billion individuals had HP infection worldwide in 2015 (Hooi et al., 2017). Multiple studies (Hatakeyama, 2004; Correa and Houghton, 2007; Peek et al., 2010; Polk and Peek, 2010; Wroblewski et al., 2010; Hatakeyama, 2014; Amieva and Peek, 2016; Navashenaq et al., 2022) have shown that HP can induce GC by stimulating intracellular inflammatory signals, modulating inflammatory and immune responses, inducing DNA damage and cellular proliferation, and generating carcinogenic bacterial toxins involved in cancer progression. Moreover, HP modifies the efficacy of anti-tumor drugs (Deng et al., 2022; Oster et al., 2022b). HP eradication can contribute to preventing the carcinogenesis and progression of GC and supporting the prevention and treatment of GC (Yan et al., 2022; Li D. et al., 2023).

Bibliometrics has been effectively applied in medical research to visualize hot topics and track the evolution of knowledge in specific fields. Several studies have carried out the bibliometric analysis of HP, such as high-cited papers in HP research (Bang et al., 2019), states and hotspots in HP research (Wang et al., 2023) and HP resistance research (Li M. et al., 2023). Over the past two decades, research on the relationship between HP and GC has steadily increased. However, there is currently a lack of quantitative investigation into this link. This paper aims to conduct a bibliometric analysis of HP/GC-related papers published in the past two decades, focusing on identifying the characteristics of the crosstalk between HP and GC. The study visualized indicators such as hotspots, topics, authors, and institutions in HP/GC research, providing researchers with a fundamental understanding of the interplay between HP and GC, and assisting scholars in better grasping the dynamic changes and trends in HP/GC-related research.

## 2 Materials and methods

### 2.1 Data source and search strategy

**Figure 1:**
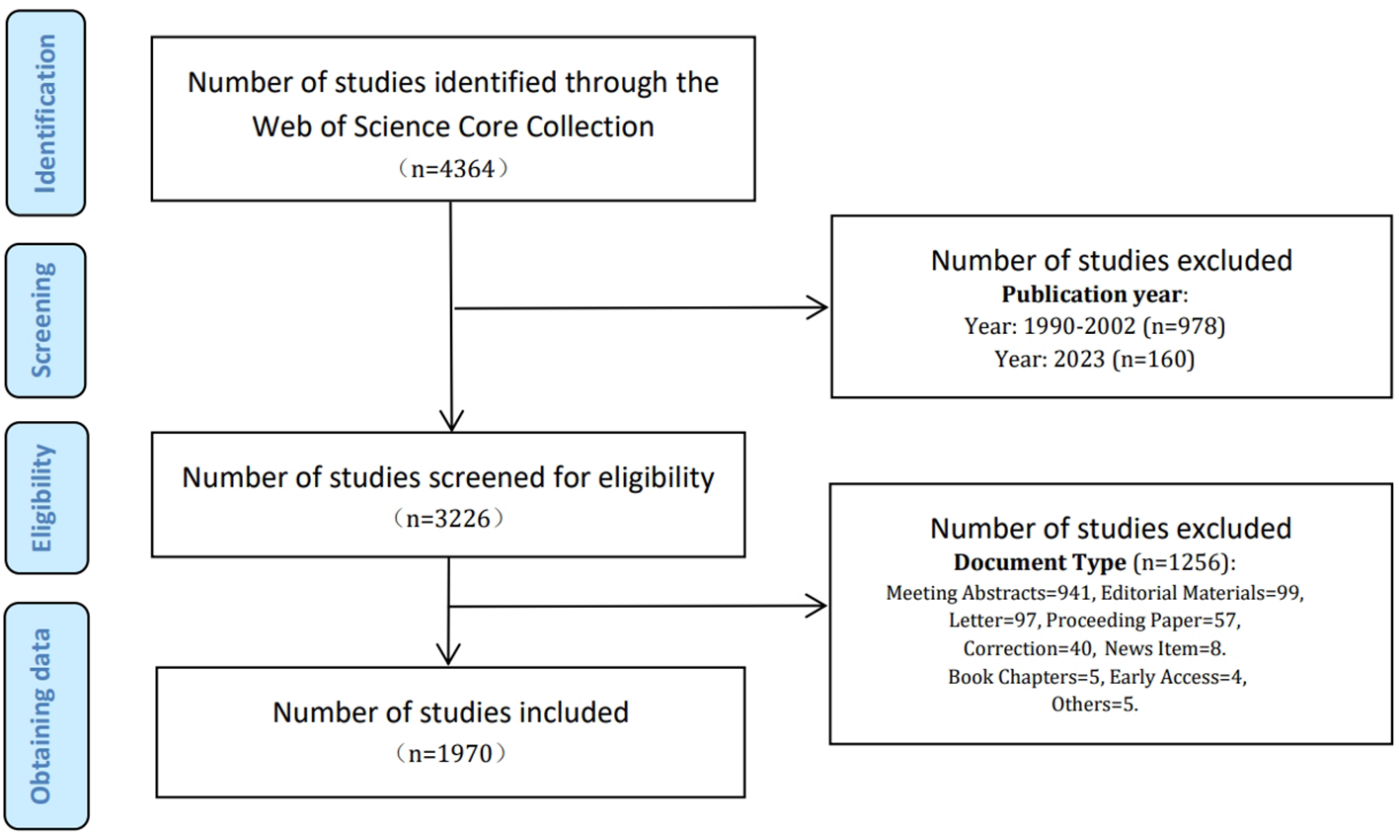
Flow chart of literature screening in HP/GC.

The WoSCC includes the most renowned and influential academic publications in natural science (Bang et al., 2019; Li M. et al., 2023; Wang et al., 2023), making it an ideal data source for our research. All search results were retrieved from the WoSCC database on October 24, 2023, using the advanced search method with the keywords “Helicobacter pylori” and “stomach cancer” and their corresponding synonyms. The synonyms for Helicobacter pylori and gastric cancer were retrieved from the MeSH Database in PubMed. The search strategy is shown in **Table 1** and **Figure 1**. The screening standards comprised (1): the publication period was from January 1, 2003 to December 31, 2022 (2): the categories included “Article” and “Review”. Finally, 1,970 papers containing 1,674 articles and 296 reviews were acquired. The search and data extraction were carried out independently by two researchers (SY and SH) and saved in text format.

**Table 1:** Search query and refinement procedure.

Set	Results	Refinement
1	4364	Step1: TI = (“Tumor*” OR “Tumour*” OR “Cancer*” OR “Neoplasia*” OR “Neoplasm*” OR “Carcinoma*” OR “Malignanc*” OR “Oncolog*” OR “Adenocarcinoma*” OR “Carcinogen*” OR “Oncogen*”) AND TS = (“Gastric” or “Stomach”) Step2: TI= (Helicobacter pylori or H.pylori) Step3: 1 AND 2
2	2456	Refined by DOCUMENT TYPES: (ARTICLES OR REVIEW ARTICLES)
3	1970	Refined by PUBLICATION YEARS: (2003–2022)

### 2.2 Data analysis and parameter query

The Bibliometrix R package and its Web applications Biblioshiny, VOSviewer, and Microsoft Excel 2019 are used for scientometric analysis. Bibliometrix (https://www.bibliometrix.org/) and VOSviewer provide a range of scientometric analysis tools for building and visualizing bibliometric networks (Bang et al., 2019; Li M. et al., 2023; Wang et al., 2023). Machine learning is used to assess the distribution of various components, such as annual production, most relevant journals/authors/affiliations/countries and their local impact by H-index or total citation (TC), and annual production over time, main funding agencies, country scientific output and collaboration network, historical direct citation network, highly cited papers and high-impact factor (IF) papers, common keywords and their cluster analysis. The impact and value of scientific papers can be assessed through citation analysis. By analyzing the number of publications in a specific field and historical direct citation networks, we can gain insight into the historical development of the field. By conducting a correlation analysis between authors and countries, one can discover potential collaborations between projects. The JCR quartile and IF were defined by the “2022 Incites Journal Citation Report”.

## 3 Results

### 3.1 Scientific output

**Figure 2:**
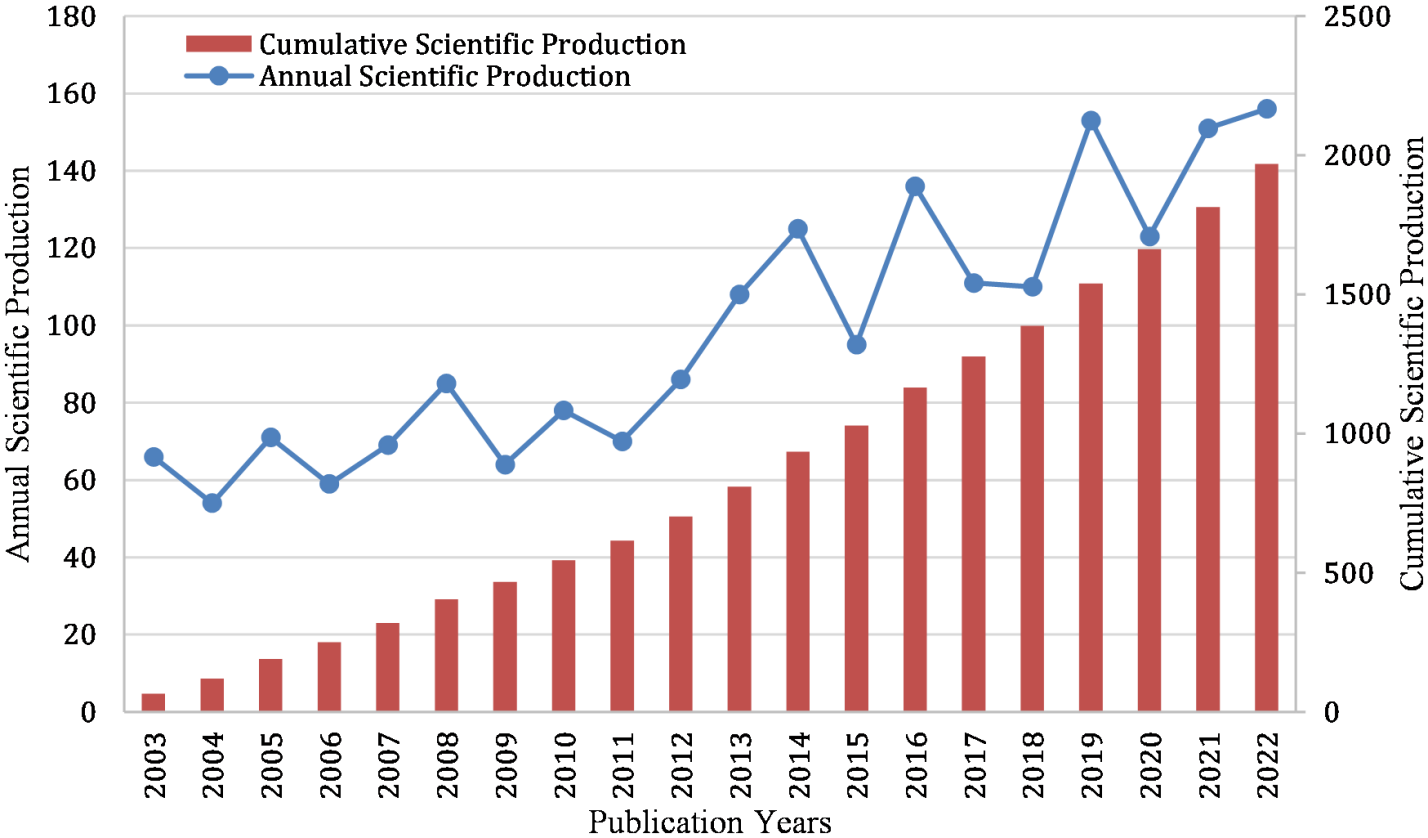
Annual and cumulative scientific output in HP/GC.

In HP/GC research, 1970 papers were retrieved. Annual production can reflect the research trend in a field. **Figure 2** lists the annual number of papers (Np) in HP/GC research from 2003 to 2022. From 2003 to 2011, the annual Np remained stable. From 2012 to 2022, the annual Np increased in waves. Before 2013, the Np was less than 100 but more than 50 per year. Since 2013 (except 2015), more than 100 papers had been published each year. In addition, the cumulative scientific output in HP/GC research was increasing from 2003 to 2022, and the growth trend remained stable.

### 3.2 Main journals output

**Figure 3:**
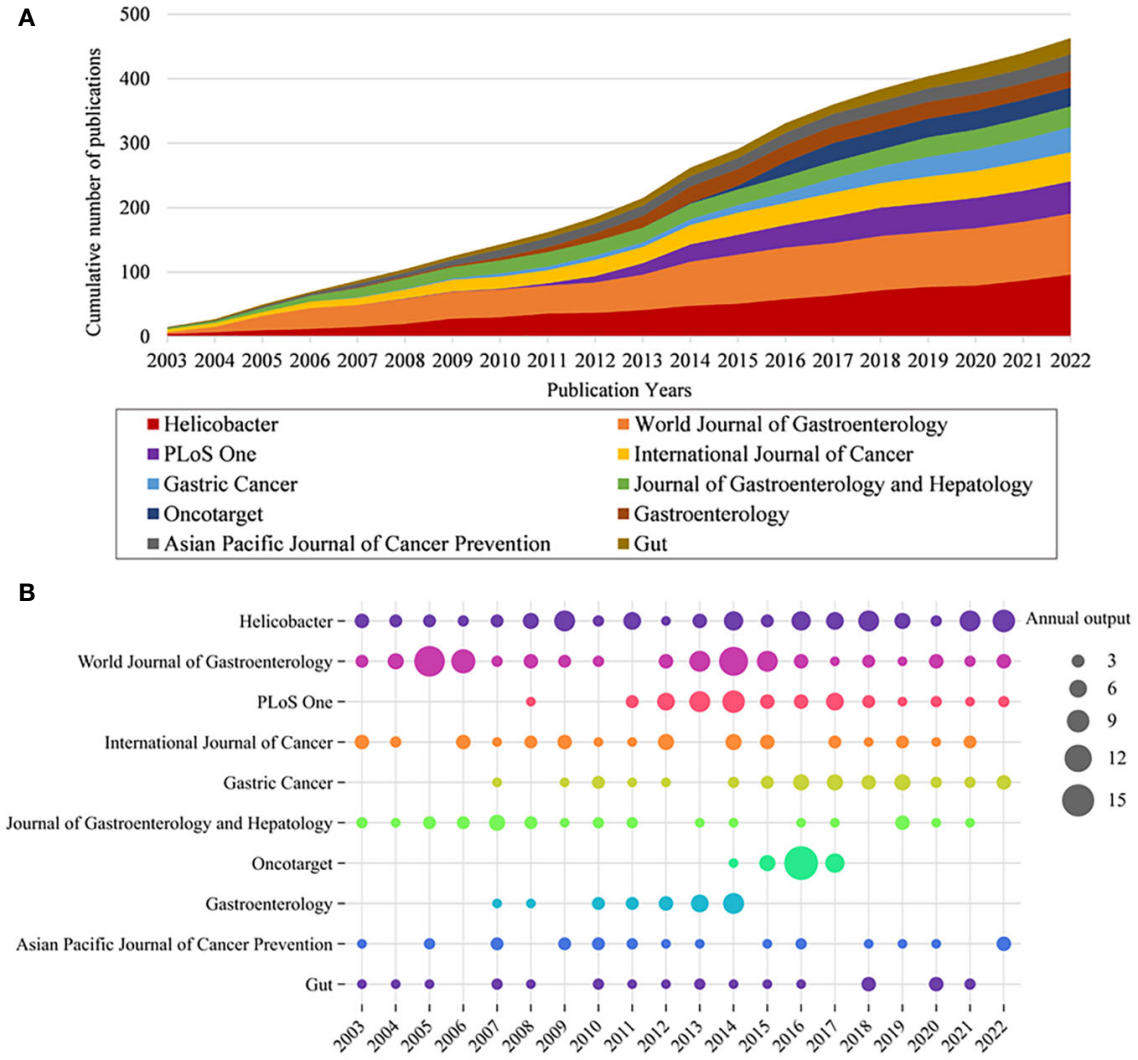
**(A)** Cumulative scientific output of the top 10 most prolific journals in HP/GC. **(B)** Annual production of the top 10 most prolific journals in HP/GC (the size of the circle represents the number, and the larger the circle, the higher the output).

The papers involved 564 journals. **Table 2** shows the top 10 journals in terms of output, with *Helicobacter* being the most productive (n = 96), followed by *World Journal of Gastroenterology* (n = 95), *PLoS One* (n = 50), *International Journal of Cancer* (n = 45), and *Gastric Cancer* (n = 39). **Figure 3A** illustrates the annual Np of the top 10 journals, with *Helicobacter* maintaining its position as the most productive journal in 2022. **Figure 3B** summarizes the cumulative Np of the top 10 journals. The Np of these journals was 463, accounting for about 24.11% of the total output, indicating their excellent production capacity. The TC indicates the significance of journals, and the H-index evaluates their academic impact. **Table 3** shows the top ten most cited journals, with *Gastroenterology* at the top, followed by *International Journal of Cancer*, *Gut*, *World Journal of Gastroenterology*, and *Helicobacter*. In terms of H-index, *International Journal of Cancer* (H-index = 30) and *World Journal of Gastroenterology* (H-index = 30) ranked the top, followed by *Helicobacter* (H-index = 28) and *PLoS One* (H-index = 24).

**Table 2:** The top 10 productive journals in HP/GC.

Rank	Journals	Np	TC	H-index	IF	JCR	Countries
1	Helicobacter	96	2390	28	4.4	Q2	UK
2	World Journal of Gastroenterology	95	2839	30	4.3	Q2	USA
3	PLoS One	50	1396	24	3.7	Q2	USA
4	International Journal of Cancer	45	3048	30	6.4	Q1	Switzerland
5	Gastric Cancer	39	1018	18	7.4	Q1	Japan
6	Journal of Gastroenterology and Hepatology	32	938	17	4.1	Q2	Australia
7	Oncotarget	29	765	16	—	—	USA
8	Gastroenterology	26	4204	23	29.4	Q1	USA
9	Asian Pacific Journal of Cancer Prevention	26	368	12	—	—	Korea
10	Gut	25	2993	23	24.5	Q1	UK

**Table 3:** The top 10 local impact journals in HP/GC.

Rank	Journals	TC	Journals	H-index
1	Gastroenterology	4204	International Journal of Cancer	30
2	International Journal of Cancer	3048	World Journal of Gastroenterology	30
3	Gut	2993	Helicobacter	28
4	World Journal of Gastroenterology	2839	PLoS One	24
5	Helicobacter	2390	Gastroenterology	23
6	PLoS One	1396	Gut	23
7	Proceedings of the National Academy of Sciences of the United States of America	1396	Gastric Cancer	18
8	Nature Reviews Cancer	1314	Journal of Gastroenterology	18
9	Cancer Letters	1198	Journal of Gastroenterology and Hepatology	17
10	Journal of Gastroenterology	1087	Alimentary Pharmacology & Therapeutics	16

### 3.3 Main authors output

**Figure 4:**
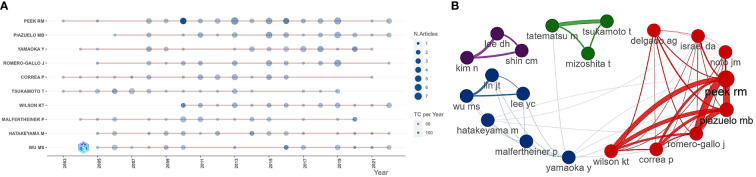
**(A)** Annual output of the top 10 most productive authors in HP/GC (the size of the circle represents output, with larger circles indicating higher output; the color depth of the circle indicates annual citations, with darker colors indicating more citations). **(B)** Co-authorship network of the 20 most productive in HP/GC (each node represents an author; each color represents a collaborative group; each line represents a coordination relationship, with thickness indicating collaboration intensity).

**Table 4** lists the ten most prolific authors and their TC and H-index. Peek Jr Richard M (n = 50), Piazuelo M Blanca (n = 32), Yamaoka Yoshio (n = 30), Romero-Gallo Judith (n = 27), and Tsukamoto Tetsuya (n = 25) took the top five places. Peek Jr Richard M had the highest Np and H-index, and the highest TC, showing his significant influence. **Figures 4A, B** respectively show the annual output of the top 10 authors and their collaboration network, with Peek Jr Richard M, Piazuelo M Blanca, Romero-Gallo Judith, Correa Pelayo and Wilson Keith T from *Vanderbilt University* having the closest cooperative relationship (a cooperative group). Moreover, other academic groups included Yamaoka Yoshio team from *Oita University*, Hatakeyama Masanori team from *University of Tokyo*, and Kim Na Young team from *Seoul National University*.

**Table 4:** The top 10 productive authors in HP/GC.

Rank	Authors	Np	TC	H-index	Affiliations	Countries
1	Peek, Richard M.	50	5,220	32	Vanderbilt University	USA
2	Piazuelo, Maria B.	32	1,643	22	Vanderbilt University	USA
3	Yamaoka, Yoshio	30	1,314	17	Oita University	Japan
4	Romero-Gallo, Judith	27	1,667	20	Vanderbilt University	USA
5	Correa, Pelayo	25	2,031	22	Vanderbilt University	USA
6	Tsukamoto, Tetsuya	25	1,242	17	Fujita Health University	Japan
7	Wilson, Keith T.	24	2,108	18	Vanderbilt University	USA
8	Malfertheiner, Peter	23	964	17	University of Munich	Germany
9	Hatakeyama, Masanori	22	2,473	19	University of Tokyo	Japan
10	Wu, Ming-Shiang	22	1,800	15	National Taiwan University	China

### 3.4 Major countries/regions and institutions output

**Figure 5:**
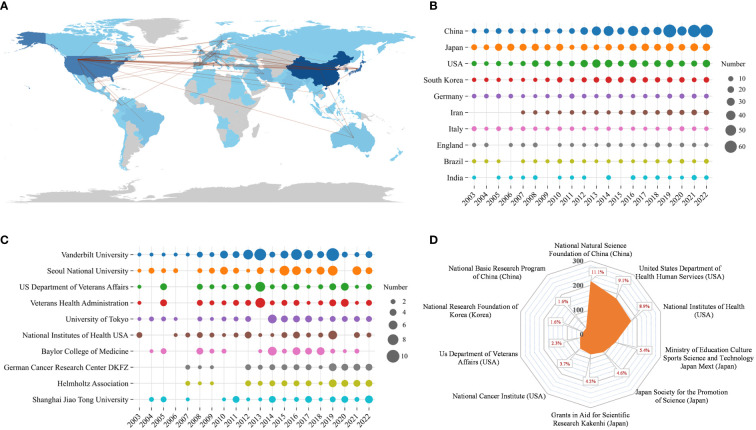
**(A)** Scientific production of various countries and their main international collaboration network in HP/GC. **(B)** Annual production of the top 10 productive countries in HP/GC. **(C)** Annual scientific production of the top 10 productive institutions in HP/GC. **(D)** The top 10 funding agencies and their source countries in HP/GC.

**Table 5** shows that most articles were published by authors from China (n = 571), Japan (n = 420), and the United States (n = 354), accounting for approximately 68.27% of the total output. **Figure 5A** presents a representation of the country scientific output and primary collaboration networks, highlighting that China had the closest ties with the USA. **Figure 5B** shows the annual Np of the top 10 countries. **Table 5** reveals the top 10 productive institutions, with *Vanderbilt University*, *Seoul National University*, *US Department of Veterans Affairs*, *Veterans Health Administration*, and *University of Tokyo* ranking among the top five. **Figure 5C** displays their annual Np. **Figure 5D** draws the main funding agencies such as *National Natural Science Foundation of China* (n =218), *United States Department of Health Human Services* (n =180), the *National Institutes of Health* (n =175), the *Ministry of Education Culture Sports Science and Technology* (n =107), and *Japan Society for The Promotion of Science* (n =90) mainly from China, the USA, and Japan, indicating that their strongly support for HP/GC research.

**Table 5:** The top 10 productive countries and institutions in HP/GC.

Rank	Countries	Np	TC	H-index	Institutions	Np	TC	H-index
1	China	571	15,837	57	Vanderbilt University	80	7,407	43
2	Japan	420	17,167	68	Seoul National University	66	1,897	26
3	USA	354	19,078	72	Us Department of Veterans Affairs	49	4797	26
4	South Korea	182	4,831	37	Veterans Health Administration	49	4,133	32
5	Germany	116	4,755	41	University of Tokyo	48	2,753	27
6	Iran	99	1,957	24	National Institutes of Health USA	42	2,184	22
7	Italy	87	3,076	34	Baylor College of Medicine	40	2,708	24
8	England	59	3,779	33	German Cancer Research Center DKFZ	39	1,090	20
9	Brazil	58	970	18	Helmholtz Association	39	1,090	20
10	India	58	1,185	20	Shanghai Jiao Tong University	39	1,129	17

### 3.5 Analysis of cited papers in HP/GC research

#### 3.5.1 Top 20 most cited articles in HP/GC research

High-cited articles are a valuable indicator in bibliometrics, reflecting extremely high academic importance and influence in a field. **Table 6** presents a list of the top 20 high-cited original research papers.

**Table 6:** The top 20 most cited original research in HP/GC.

Rank	Title	First author	Year	Journals	IF	JCR	TC
1	Helicobacter pylori eradication to prevent gastric cancer in a high-risk region of China -: A randomized controlled trial	Wong, BCY	2004	JAMA-J. Am. Med. Assoc.	120.7	Q1	1064
2	Effect of eradication of Helicobacter pylori on incidence of metachronous gastric carcinoma after endoscopic resection of early gastric cancer: an open-label, randomised controlled trial	Fukase, K	2008	Lancet	168.9	Q1	883
3	Global burden of gastric cancer attributable to Helicobacter pylori	Plummer, M	2015	Int. J. Cancer	6.4	Q1	575
4	High levels of aberrant DNA methylation in Helicobacter pylori: Infected gastric mucosae and its possible association with gastric cancer risk	Maekita, T	2006	Clin. Cancer Res.	11.5	Q1	498
5	Transgenic expression of Helicobacter pylori CagA induces gastrointestinal and hematopoietic neoplasms in mouse	Ohnishi, N	2008	Proc. Natl. Acad. Sci. U. S. A.	11.1	Q1	421
6	Progression of chronic atrophic gastritis associated with Helicobacter pylori infection increases risk of gastric cancer	Ohata, H	2004	Int. J. Cancer	6.4	Q1	392
7	Activation of β-catenin by carcinogenic Helicobacter pylori	Franco, AT	2005	Proc. Natl. Acad. Sci. U. S. A.	11.1	Q1	381
8	Helicobacter pylori Therapy for the Prevention of Metachronous Gastric Cancer	Choi, IJ	2018	N. Engl. J. Med.	158.5	Q1	379
9	Fifteen-Year Effects of Helicobacter pylori, Garlic, and Vitamin Treatments on Gastric Cancer Incidence and Mortality	Ma, JL	2012	J. Natl. Cancer Inst.	10.3	Q1	314
10	Predicting the development of gastric cancer from combining Helicobacter pylori antibodies and serum pepsinogen status: a prospective endoscopic cohort study	Watabe, H	2005	Gut	24.5	Q1	313
11	A new Helicobacter pylori vacuolating cytotoxin determinant, the intermediate region, is associated with gastric cancer	Rhead, JL	2007	Gastroenterology	29.4	Q1	294
12	Long-term proton pump inhibitors and risk of gastric cancer development after treatment for Helicobacter pylori: a population-based study	Cheung, KS	2018	Gut	24.5	Q1	271
13	Helicobacter pylori infection and gastric atrophy: Risk of adenocarcinoma and squamous-cell carcinoma of the esophagus and adenocarcinoma of the gastric cardia	Ye, WM	2004	JNCI-J. Natl. Cancer Inst.	10.3	Q1	262
14	Opposing risks of gastric cardia and noncardia gastric adenocarcinomas associated with Helicobacter pylori seropositivity	Kamangar, F	2006	JNCI-J. Natl. Cancer Inst.	10.3	Q1	253
15	Lack of Commensal Flora in Helicobacter pylori-Infected INS-GAS Mice Reduces Gastritis and Delays Intraepithelial Neoplasia	Lofgren, JL	2011	Gastroenterology	29.4	Q1	244
16	Promoter methylation of E-cadherin gene in gastric mucosa associated with Helicobacter pylori infection and in gastric cancer	Chan, AOO	2003	Gut	24.5	Q1	244
17	The benefit of mass eradication of Helicobacter pylori infection: a community-based study of gastric cancer prevention	Lee, YC	2013	Gut	24.5	Q1	240
18	Early Helicobacter pylori Eradication Decreases Risk of Gastric Cancer in Patients With Peptic Ulcer Disease	Wu, CY	2009	Gastroenterology	29.4	Q1	217
19	Regulation of gastric carcinogenesis by helicobacter pylori virulence factors	Franco, AT	2008	Cancer Res.	11.2	Q1	212
20	Carcinogenic bacterial pathogen Helicobacter pylori triggers DNA double-strand breaks and a DNA damage response in its host cells	Toller, IM	2011	Proc. Natl. Acad. Sci. U. S. A.	11.1	Q1	211

In clinical research, several studies (Wong et al., 2004; Fukase et al., 2008; Wu et al., 2009; Lee et al., 2013; Choi et al., 2018) published in world-famous journals have shown that eradication of HP can prevent the occurrence of GC and significantly decrease the development of GC. Among them, two studies (Fukase et al., 2008; Choi et al., 2018) showed that patients with early GC treated with HP had a lower incidence of metachronous GC. A longitudinal cohort study (Ohata et al., 2004) showed that there is a strong positive correlation between the degree of gastritis caused by HP and the development of cancer, especially for intestinal GC, indicating that severe gastritis with extensive intestinal metaplasia is a major risk factor for GC. A prospective case-control study (Kamangar et al., 2006) showed that HP was an important risk factor for non-cardia GC, but negatively correlated with the risk of cardia GC. It is speculated that the decrease in the prevalence of HP may lead to a decrease in the incidence of non-cardia cancer and an increase in the incidence of cardia cancer in Western countries. Interestingly, a study (Ye et al., 2004) showed that HP infection may be not related to the risk of gastric cardia adenocarcinoma but reduce the risk of esophageal adenocarcinoma.

Moreover, in terms of diagnosis, the combination of serum pepsinogen and anti-HP antibody provides a good predictive marker for the development of GC (Watabe et al., 2005). Immunoblotting is more sensitive for detecting anti-HP antibodies than ELISA (Plummer et al., 2015). In treatment, an intervention trial (Ma et al., 2012) showed that garlic and vitamin treatments were associated with non-statistically significant reductions in GC incidence and mortality. On the contrary, a multicenter study (Cheung et al., 2018) showed that long-term use of proton pump inhibitors was associated with an increased risk of GC even after HP eradication. Furthermore, an animal study (Ohnishi et al., 2008) showed that transgenic expression of HP CagA induced gastrointestinal and hematopoietic tumors. A study (Franco et al., 2005) showed that β-catenin nuclear accumulation was increased in gastric epithelium collected from gerbils infected with HP carcinogenic strains. A comparative study (Maekita et al., 2006) showed that HP infection can induce CpG island methylation to varying degrees, and the methylation level of specific CpG islands seems to reflect the cancer risk of HP-negative individuals. In addition, a study (Rhead et al., 2007) has shown that the vacuolating cytotoxin A (VacA) is the main determinant of HP virulence, and the VacA i region is an important determinant of the virulence of HP and the best independent marker of VacA-related pathogenicity. A basic study (Lofgren et al., 2011) showed that the lack of commensal microbiota in HP-infected INS-GAS mice can reduce gastritis and delay intraepithelial neoplasia.

#### 3.5.2 Top 10 most cited reviews in HP/GC research

A review can provide timely guidance for scholars with a large amount of information, including research development, existing problems, and future trends. **Table 7** shows the top 10 most cited reviews, mainly from *Nature Reviews Cancer* (n = 2) and *Gastroenterology* (n = 4). Two review articles (Hatakeyama, 2004; Hatakeyama, 2014) outlined the oncogenic mechanisms of the HP CagA protein and the signals emitted by CagA abnormalities integrated into direct carcinogenic damage and genetic instability. CagA-mediated gastric carcinogenesis is carried out through a hit-and-run mechanism. In the process of long-term infection with CagA-positive HP, the carcinogenic effect of CagA is replaced by a series of genetic or epigenetic changes compiled in precancerous lesions. Two meta-analyses (Fuccio et al., 2009; Lee et al., 2016) showed that HP eradication treatment may reduce the risk of GC. Several review articles (Correa and Houghton, 2007; Polk and Peek, 2010; Wroblewski et al., 2010; Wang et al., 2014; Amieva and Peek, 2016) discussed the various factors of HP-induced GC, including host immune response, polymorphism, changes in the apical junction complex, strain-specific bacterial components, specific host-microbe interactions, and the influence of environmental factors such as dietary components and essential micronutrients, as well as the gastrointestinal microbiota. A review (Graham, 2015) described the mechanism of HP in the development of GC, reliable treatment and possible benefits.

**Table 7:** The top 10 most cited reviews in HP/GC.

Rank	Title	First author	Year	Journals	IF	JCR	TC
1	Helicobacter pylori and Gastric Cancer: Factors That Modulate Disease Risk	Wroblewski, LE	2010	Clin. Microbiol. Rev.	36.8	Q1	896
2	Helicobacter pylori: gastric cancer and beyond	Polk, DB	2010	Nat. Rev. Cancer	78.5	Q1	742
3	Oncogenic mechanisms of the Helicobacter pylori CagA protein	Hatakeyama, M	2004	Nat. Rev. Cancer	78.5	Q1	572
4	Pathobiology of Helicobacter pylori-Induced Gastric Cancer	Amieva, M	2016	Gastroenterology	29.4	Q1	501
5	Carcinogenesis of Helicobacter pylori	Correa, P	2007	Gastroenterology	29.4	Q1	493
6	Association Between Helicobacter pylori Eradication and Gastric Cancer Incidence: A Systematic Review and Meta-analysis	Lee, YC	2016	Gastroenterology	29.4	Q1	490
7	Helicobacter pylori-induced gastric inflammation and gastric cancer	Wang, F	2014	Cancer Lett.	9.7	Q1	458
8	Helicobacter pylori CagA and Gastric Cancer: A Paradigm for Hit-and-Run Carcinogenesis	Hatakeyama, M	2014	Cell Host Microbe	30.3	Q1	313
9	Meta-analysis: Can Helicobacter pylori Eradication Treatment Reduce the Risk for Gastric Cancer?	Fuccio, L	2009	Ann. Intern. Med.	39.2	Q1	285
10	Helicobacter pylori Update: Gastric Cancer, Reliable Therapy, and Possible Benefits	Graham, DY	2015	Gastroenterology	29.4	Q1	269

### 3.6 High-IF papers in HP/GC research

High-impact Factor (IF) papers are considered a crucial metric for assessing the research quality and influence of scholars, playing a pivotal role in advancing the development of a discipline. High-IF papers tend to captivate the attention and citation of international peers. As high-cited papers tend to be published in high-IF journals, we searched for the articles in high-IF (IF>30) journals (**Table 8**).

**Table 8:** The high impact factors papers in HP/GC.

No.	Doi	Type	Journals	First Author	Year	TC
1	10.1001/jama.291.2.187	Article	JAMA-J. Am. Med. Assoc.	Wong, BCY	2004	1064
2	10.1128/CMR.00011-10	Review	Clin. Microbiol. Rev.	Wroblewski, LE	2010	896
3	10.1016/S0140-6736(08)61159-9	Article	Lancet	Fukase, K	2008	883
4	10.1038/nrc2857	Review	Nat. Rev. Cancer	Polk, DB	2010	742
5	10.1038/nrc1433	Review	Nat. Rev. Cancer	Hatakeyama, M	2004	572
6	10.1056/NEJMoa1708423	Article	N. Engl. J. Med.	Choi, IJ	2018	379
7	10.1016/j.chom.2014.02.008	Review	Cell Host Microbe	Hatakeyama, M	2014	313
8	10.7326/0003-4819-151-2-200907210-00009	Review	Ann. Intern. Med.	Fuccio, L	2009	285
9	10.1136/bmj.g3174	Review	BMJ-British Medical Journal	Ford, AC	2014	219
10	10.1056/NEJMoa1909666	Article	N. Engl. J. Med.	Choi, IJ	2020	182
11	10.1152/physrev.00039.2009	Review	Physiol. Rev.	Peek, RM	2010	162
12	10.1016/j.chom.2012.10.014	Article	Cell Host Microbe	Tsugawa, H	2012	161
13	10.1136/bmj.l5016	Article	BMJ-British Medical Journal	Li, WQ	2019	129
14	10.1200/JCO.2009.26.0695	Article	J. Clin. Oncol.	Wu, CY	2010	111
15	10.1016/j.chom.2012.05.010	Article	Cell Host Microbe	Hayashi, T	2012	110
16	10.1093/annonc/mdr384	Article	Ann. Oncol.	González, CA	2012	85
17	10.1016/S1470-2045(06)70586-1	Article	Lancet Oncol.	Meimarakis, G	2006	82
18	10.1093/annonc/mdi184	Article	Ann. Oncol.	Ruzzo, A	2005	56
19	10.1016/S2468-2667(21)00164-X	Article	Lancet Public Health	Yang, L	2021	50
20	10.1016/j.chom.2021.04.006	Article	Cell Host Microbe	Imai, S	2021	44
21	10.1093/annonc/mdn055	Article	Ann. Oncol.	Prasad, KN	2008	24

In original articles, there were 14 papers were extracted. Among them, *Cell Host & Microbe* (n = 3), Annals *of Oncology* (n = 3), and *Lancet* and its sub-journals (n = 3) had the most publications, followed by *New England Journal of Medicine* (n = 2), *BMJ* (n = 1), *JAMA* (n = 1), and *Journal of Clinical Oncology* (n = 1). Some high-cited papers have been described above (Wong et al., 2004; Fukase et al., 2008; Choi et al., 2018). In addition, several research found that HP eradication therapy can reduce the risk of GC in HP-infected patients with a family history of GC in first-degree relatives (Choi et al., 2020). HP treatment was associated with a statistically reduced risk of GC death and incidence of GC (Li et al., 2019). The screening and eradication of HP can reduce the burden of GC in high-risk populations in Chinese adults (Yang et al., 2021). Moreover, a prospective study (Meimarakis et al., 2006) showed that HP may be seem as a prognostic indicator after curative resection of gastric carcinoma. Three articles (Hayashi et al., 2012; Tsugawa et al., 2012; Imai et al., 2021) analyzed the carcinogenic mechanism of HP CagA, including the inhibition of autophagic degradation, the pathogenic signal enhancement, and genomic instability. The Eurogast-EPIC study found that 93.2% of GC patients were positive for HP infection (González et al., 2012). The interleukin-1B gene (IL-1B), interleukin-1 receptor antagonist gene (IL-1RN), and PPARγ Pro12Ala polymorphism act in HP-associated gastric adenocarcinoma (Ruzzo et al., 2005; Prasad et al., 2008). Regular use of nonsteroidal anti-inflammatory drugs may be a feasible method to prevent GC, especially in patients with HP infection (Wu et al., 2010).

In review articles, total seven high-IF reviews were published between 2004 and 2019, mainly from *Nature Reviews Cancer* (n = 2), *BMJ* (n = 1), *Clinical Microbiology Reviews* (n = 1), *Cell Host & Microbe* (n = 1), *Physiological Reviews* (n = 1), and *Annals of Internal Medicine* (n = 1). Among them, several high-cited review articles (Hatakeyama, 2004; Polk and Peek, 2010; Wroblewski et al., 2010; Hatakeyama, 2014) had described that the relationship between bacterial virulence factors VacA and CagA protein, outer membrane proteins, inflammation, host immune response, environmental factors and HP-mediated GC. HP eradication treatment may reduce the risk of GC (Fuccio et al., 2009). Apart from the above high-cited reviews, there were three high-IF reviews worthy of attention. Peek RJ et al (Peek et al., 2010). further depicted that the role of host innate immune system including gastric epithelial cells and immune cells in HP-induced GC. A meta-analysis (Ford et al., 2014) showed that HP eradication therapy may reduce the incidence of GC in healthy asymptomatic infected Asian individuals.

### 3.7 Keywords in HP/GC research

#### 3.7.1 High-frequency keywords

**Figure 6:**
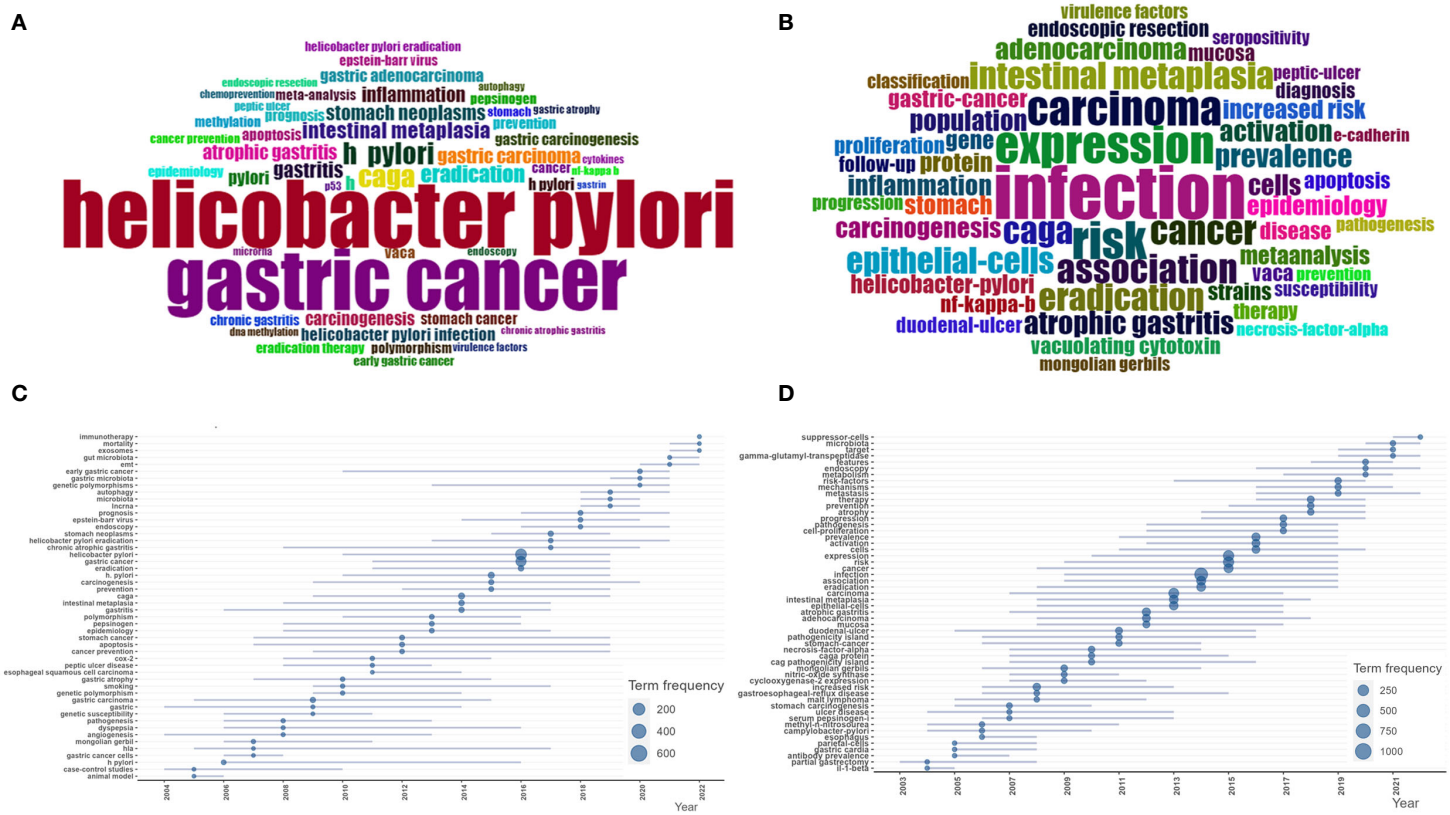
**(A)** Common author keywords in HP/GC. **(B)** Common keywords Plus in HP/GC. **(C)** Common author keywords evolution trends in HP/GC. **(D)** Common keywords Plus evolution trend in HP/GC.

We examine keywords to pick out the hot topics in HP/GC research. In this paper, a total of 5,811 keywords included 3,176 author’s keywords and 2,635 keywords plus were acquired. The common author’s keywords (**Figure 6A**) included “*Helicobacter pylori*”, “gastric cancer”, “CagA”, “diet”, “eradication”, “intestinal metaplasia”, “gastritis”, “stomach neoplasms”, “gastric carcinoma”, “atrophic gastritis”, “inflammation”, “carcinogenesis”, “gastric adenocarcinoma”, “VacA”, “gastric carcinogenesis”, “apoptosis”, “stomach cancer”, “prognosis”, “prevention”, “meta-analysis”, “cancer”, etc. The common keywords plus (**Figure 6B**) included “infection”, “expression”, “risk”, “carcinoma”, “association”, “cancer”, “CagA”, “eradication”, “intestinal metaplasia”, “epithelial-cells”, “prevalence”, “atrophic gastritis”, “activation”, “adenocarcinoma”, “population”, “cells”, “inflammation”, “carcinogenesis”, etc.

#### 3.7.2 Time series analysis of keywords

Keyword time series analysis in the bibliometric analysis can observe the changes of keyword frequency and co-occurrence relationship over time, so as to reveal the development trend and hot spot changes of research topics. By means of trend topic module in the biblioshiny of Bibliometrix, we analyze the time evolution of keywords. As we can see in **Figures 6C, D**, the hot author’s keywords in recent years include “autophagy”, “microbiota”, “lncRNA”, “early gastric cancer”, “EMT”, “exosomes”, “mortality”, “gut microbiota”, “immunotherapy”, and so on. The hot keywords plus include “mechanisms”, “metastasis”, “features”, “endoscopy”, “metabolism”, “microbiota”, “target”, “gamma-glutamyl-transpeptidase”, “suppressor-cells”, and so on.

#### 3.7.3 Cluster analysis of keywords

**Figure 7:**
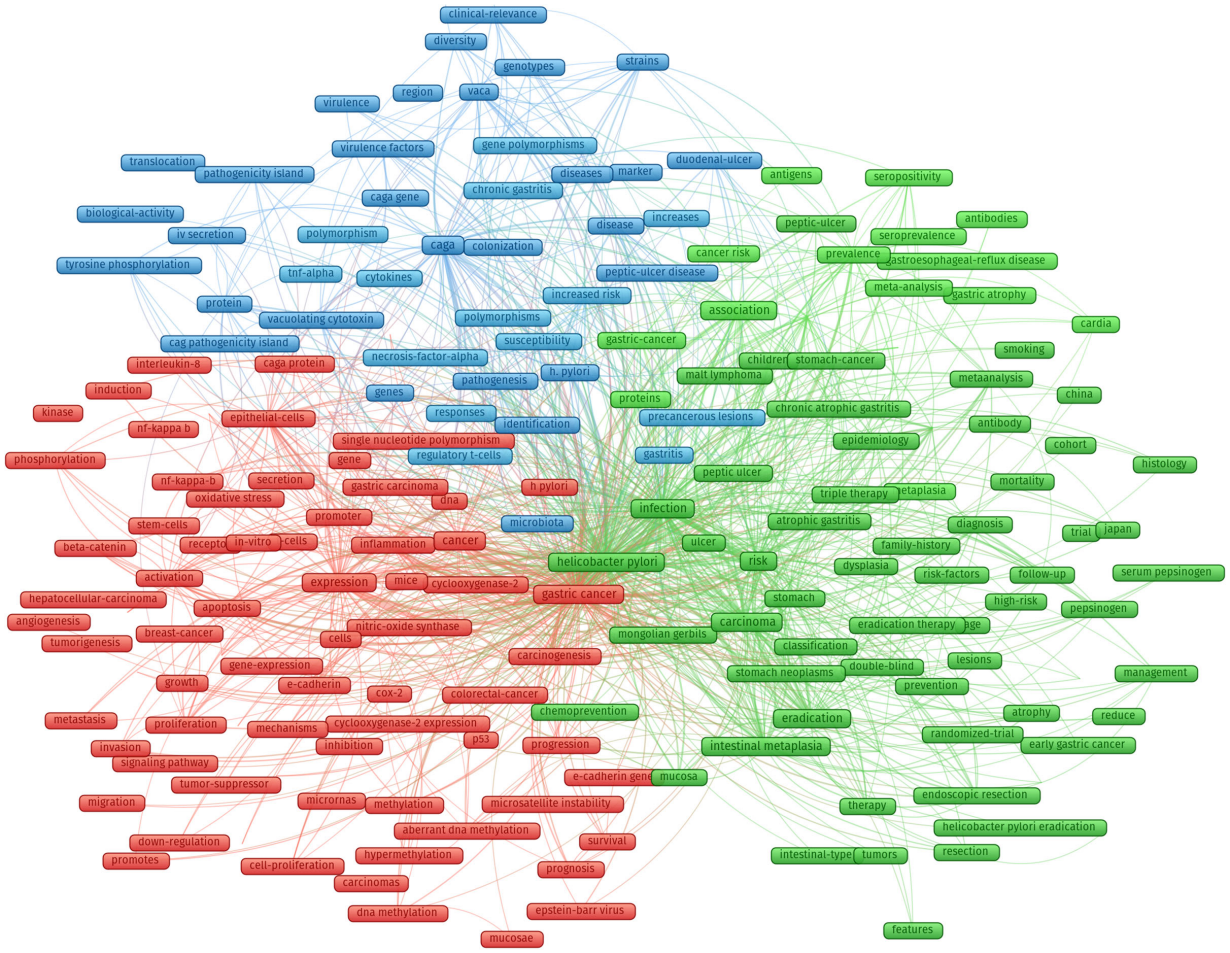
Cluster analysis of all common keywords in HP/GC (different colors mean different clusters, the size of the circle indicates the frequency of occurrence of keywords).

According to the extracted keywords, the co-occurrence network between them is constructed, and the relationship between them is established according to the situation that the keywords appear in the same literature at the same time. Based on the co-occurrence keywords, the cluster analysis was conducted to assess the links of the keywords, and the cluster analysis results were shown in **Figure 7**.

Cluster 1 (green nodes) concerned the links between HP and digestive system disease (such as chronic atrophic gastritis, MALT lymphoma, peptic ulcer, intestinal metaplasia, dysplasia, gastric cancer). HP may cause gastric mucosal inflammation in patients, such as chronic superficial gastritis, chronic atrophic gastritis. It can also cause peptic ulcer, including gastric ulcer, duodenal ulcer, etc. With the severity of the disease, HP will gradually destroy the gastrointestinal wall, causing the onset of GC.

Cluster 2 (blue nodes) focused on the pathogenesis of HP-induced GC, especially HP-related virulence factors, including CagA and its pathogenicity island (CagPAI), IV secretion, tyrosine phosphorylation, VacA and gene polymorphism, and precancerous lesions. CagA and VacA are the main virulence factors of HP. CagA is an outer membrane protein with strong immunogenicity, which is present in the cytoplasm of HP and eventually transported into host cells through Type IV secretion system. VacA is a vacuolating toxin produced by HP, which can induce vacuolation and inhibit immune response.

Cluster 3 (red nodes) focused on the mechanisms by which HP affects GC, especially host factors, including inflammation (nf-kappa-b, interleukin-8), β-catenin, E-cadherin, immunity (such as regulatory T cells and intestinal epithelial cells), gene-expression, stem cells, nitric-oxide synthase, p53, COX2 and oxidative stress. Inflammation and immune factors play a key role in the pathogenesis of HP infection. Inflammatory response is one of the important causes of gastric mucosal injury, and also promotes the proliferation and apoptosis of gastric mucosal cells, and ultimately forms tumors.

## 4 Discussions

In 1994, the World Health Organization (WHO) classified HP as type I carcinogen for GC. Since then, HP/GC research has received increasing attention from scholars. Over the past two decades, given the growing understanding of HP and evidence that HP is a modulator that can influence the occurrence and progression of GC, and alter the outcome of GC treatment, numerous studies have been conducted on the link between HP and GC. Therefore, we mainly focus on literature from the past two decades to better track the status and trends in HP/GC research.

### 4.1 Analysis of document issuance in HP/GC research

From the annual Np view, from 2003 to 2011, HP/GC research gradually gained steady attention, and the number of articles published each year was more than 50. Since 2011, the research had gradually increased and the annual number of publications was more than 100. Regarding the journals, our study showed that *Helicobacter*, *World Journal of Gastroenterology* and *PLoS One* ranked in the top three in the Np, *International Journal of Cancer* and *World Journal of Gastroenterology* had the highest H-index, and the *Gastroenterology* had the most TC. *Helicobacter* is a broad-caliber journal that covers the full spectrum of HP research, and promotes communication between the fields of gastroenterology, microbiology, vaccine development, and laboratory animal science. In addition, it is worth noting that the top 20 high-cited and high-IF articles were published mainly in *NEJM* (Choi et al., 2018; Choi et al., 2020; Usui et al., 2023) and *Lancet and its sub-journals* (Meimarakis et al., 2006; Fukase et al., 2008; Yang et al., 2021), followed by *JAMA* (Wong et al., 2004), *BMJ* (Li et al., 2019), *Gut* (Watabe et al., 2005; Lee et al., 2013; Cheung et al., 2018; Oster et al., 2022b), *Gastroenterology* (Wu et al., 2009; Yan et al., 2022; Li D. et al., 2023), and so on. They mainly focused more on clinical research, while *PNAS* (Franco et al., 2005; Ohnishi et al., 2008) focused more on basic experimental research. These prestigious journals are more likely to publish top studies in the next year. *Nature Reviews Cancer* (Hatakeyama, 2004; Polk and Peek, 2010; Thrift et al., 2023) and *Gastroenterology* (Correa and Houghton, 2007; Graham, 2015; Amieva and Peek, 2016; Lee et al., 2016) had the most influential reviews, showing that them can publish more reviews in the next year. These original articles and review articles deserve the attention of researchers, as they often represent significant research achievements in the field, through which we can gain insight into the latest advances in the current research area and identify research directions.

These papers came mainly from China and Japan, followed by the United States, Korea and Germany. China had the largest Np, followed by Japan, which may be due to high incidence of HP (Hooi et al., 2017) and GC (the incidence of GC in China accounts for almost half of the world) (Etemadi et al., 2020) and the high attention and support of the countries. The top 10 institutions were from China, the United States and Japan, showing their good scientific productivity. In China, *Peking University* and *Shanghai Jiao Tong University* published the most papers. *University of Tokyo* and *Oita University* in Japan, and *Vanderbilt University* and *US Department of Veterans Affairs* in the United States made important contributions to HP/GC research. Most of the top 10 authors were from *Vanderbilt University* and *Seoul National University*, which are the world-class research universities. Peek Richard M, a gastroenterologist from *Vanderbilt University*, had most Np and the highest H-index and TC, and had devoted himself to the study of HP, especially the tumorigenesis and pathobiology of HP-induced GC such as activation of β-catenin, virulence factors, innate immunity, microRNAs, iron deficiency and regulation of p53 (Peek et al., 2010; Polk and Peek, 2010; Wei et al., 2010; Wroblewski et al., 2010; Noto et al., 2013; Amieva and Peek, 2016). Latterly, he increasingly focused on the role of gastric microbiome (Noto and Peek, 2017), hydrogen metabolism (Wang et al., 2016) and bile acid metabolism (Noto et al., 2022) in the HP-induced GC. Yamaoka Yoshio from *Oita University* had long been engaged in HP virulence factors and the link between GC and HP infection in East Asian populations (Binh et al., 2017; Sugimoto et al., 2020). Piazuelo M Blanca and Romero-Gallo Judith from *Vanderbilt University* had published many high-cited papers (working closely with Peek Richard M) (Wei et al., 2010; Noto et al., 2013), and the former found the nutraceutical electrophile scavenger 2-hydroxybenzylamine can attenuate GC development caused by HP (Gobert et al., 2023).

### 4.2 Historical cited papers in HP/GC research

The historiography analysis revealed several classic papers (deserve special attention), annotated with their local citation score (LCS) and global citation score (GCS) in **Supplementary Material S1**.

In 2004, Wong et al. (2004) showed that eradication of HP significantly reduced the development of CG in HP carriers without precancerous lesions. Hatakeyama (2004) et al. described the oncogenic mechanisms of the HP CagA protein. In 2005, Franco et al. (2005) indicated that HP-induced dysregulation of beta-catenin-dependent pathways may explain the augmentation in HP-induced GC. In 2006, Kamangar et al. (2006) further showed that HP is a strong risk factor for non-cardia GC but is inversely associated with the risk of cardia GC. In 2007, Correa (Correa and Houghton, 2007) reviewed the carcinogenesis of HP, which begins with early inflammation, progresses through metaplasia and atypical hyperplasia, and ultimately leads to cancer development. In 2008, Fukase et al. (2008) found that HP should be eradicated after endoscopic resection of early GC to prevent the development of metachronous GC. Ohnishi et al. (2008) found that HP CagA protein transgenic expression can induce gastrointestinal and hematopoietic tumors in mice. In 2009, a meta-analysis (Fuccio et al., 2009) showed that HP eradication therapy may reduce the risk of GC. Wroblewski et al. (2010) discussed that the main virulence determinants of HP strains and the correlation between these factors and different clinical outcomes after HP infection. In 2010, Polk et al (Polk and Peek, 2010). summarized the possible mechanism of HP leading to GC. A review (Wroblewski et al., 2010) showed that HP virulence factors, host factors, and environmental factors can affect the occurrence and development of GC. In 2012, Ma et al. (2012) showed that HP treatment significantly reduces the incidence of GC. Inversely, Maehata et al. (2012) found that eradication of HP did not reduce the incidence of metachronous GC. HP should be eradicated before the progression of gastric mucosal atrophy. In 2013, Lee et al. (2013) showed that HP eradication led to a significant reduction in gastric atrophy, but at the cost of increased esophagitis. In 2014, Wang et al. (2014) described the pathophysiological mechanism of HP-induced gastric inflammation and GC. In 2016, Amieva et al (Amieva and Peek, 2016). further described the pathology of HP-induced GC. A meta-analysis (Lee et al., 2016) showed that eradication of HP can effectively reduce the incidence of GC, and the protective effect is greater in individuals with a higher baseline risk of GC. In 2018, a study (Choi et al., 2018) showed that patients with early GC receiving HP treatment had a lower incidence of metachronous GC, and the degree of gastric atrophy was more improved than baseline.

### 4.3 Research status and hotspots in HP/GC research

This paper found that the current hot topics were mainly concentrated in the effect of HP on tumorigenesis and treatment of GC and the possible mechanisms of HP involved in GC.

#### 4.3.1 Effect of HP on gastric tumorigenesis and progression

Some studies showed that HP can promote the progression and carcinogenesis of GC. In a study of 114 histologically confirmed GC cases from eastern Libya, the total HP infection rate was 63.2%, particularly for intestinal-type gastric adenocarcinoma (71.7%) (Elzouki et al., 2012). The infection rate of HP in the GC group was significantly higher than that in the non-GC group, and patients with HP infection had a higher risk of non-cardia GC than those without infection (Binh et al., 2017). There is a strong positive correlation between the degree of gastritis caused by HP and the development of GC, especially in intestinal gastritis, and the progression of chronic atrophic gastritis with HP infection increased the risk of GC (Ohata et al., 2004). Correa outlines the histological progression of HP infection from the precancerous cascade to cancer (Correa and Houghton, 2007). Furthermore, HP is a strong risk factor for non-cardia GC but is inversely associated with the risk of cardia GC (Kamangar et al., 2006). HP infection after endoscopic resection may increase the risk of metachronous GC development (Kim et al., 2014).

Consequently, eradication of HP significantly reduced the risk of developing GC in patients without precancerous lesions, providing additional evidence that HP affects the early stages of GC (Wong et al., 2004). In the East Asian population at high risk of GC, HP eradication effectively reduced the risk of GC regardless of the history of cancer (Sugimoto et al., 2020). Early HP eradication is associated with decreased risk of GC in patients with peptic ulcer diseases (Wu et al., 2009). Several meta-analyses (Fuccio et al., 2009; Lee et al., 2016) also showed that eradication of HP infection was associated with a reduced incidence of GC. In addition, a 2020 double-blind study (Choi et al., 2020) reported that HP eradication therapy can reduce the risk of GC in HP-infected patients with a family history of GC in first-degree relatives. For metachronous GC, a study (Choi et al., 2018) showed that patients with early GC who received HP eradication therapy had a lower incidence of metachronous GC and greater improvement in gastric atrophy grading than patients treated with placebo. A meta-analysis demonstrated that HP eradication can reduce the occurrence of metachronous GC in patients who underwent endoscopic resection (Yoon et al., 2014).

#### 4.3.2 Pathological mechanisms of HP-induced GC

HP-induced GC is the result of a complex interaction between bacterial virulence factors, the host inflammatory response and environmental impact (Noto et al., 2013). HP contributes to gastric carcinogenesis through bacterial virulence factors and metabolites, chronic inflammation, host immunity, barrier disruption, alterations of cell proliferation and cell invasion and apoptosis, and so on (Peek et al., 2010; Wang et al., 2014).

First of all, CagA and VacA are the main virulence factors of HP. CagA and VacA can trigger inflammation and carcinogenesis (Hatakeyama, 2004). Cag A enters gastric epithelial cells via the bacterial type IV secretion system. Notably, CagA is known for its variation, which may influence the potential of different HP strains to promote GC (Hatakeyama, 2014). HP CagA triggers BRCAness to induce genomic instability, which may underlie the development of bacterial GC (Imai et al., 2021). Phosphorylated activated CagA interacts with a variety of host proteins in target cells and continuously activates the abnormal expression of multiple carcinogenic signaling pathways (Yong et al., 2015). Likewise, the risk of gastric cardia and non-cardia adenocarcinoma is much higher in CagA-positive HP infection than in CagA-negative infection (Carlosama-Rosero et al., 2021). VacA can not only induce vacuolization of gastric epithelial cells, but also stimulate apoptosis (Polk and Peek, 2010). Capurro et al. (2019) found that VacA targets the lysosomal calcium channel TRPML1 to disrupt the lysosomal transport, and thereby hijack the lysosomal and autophagy pathways, allowing HP to escape the role of antibiotics, and ultimately survive in the stomach and continuously stimulate host cells.

In addition, inflammation promotes the progression of HP-associated GC, which is supported by the higher incidence of GC in gastritis patients, especially in patients with intestinal metaplasia and dysplasia. HP-associated GC occurs primarily through the inflammatory-cancer pathway. Specifically, HP-induced inflammation leads to a high renewal rate of gastric endothelial cells, high levels of reactive oxygen species and nitrogen in the microenvironment, and an increased likelihood for DNA damage and somatic mutations (Graham, 2015). HP infection can up-regulate many pro-inflammatory cytokines, such as IL-1β, IL-6, IL-8, TNF-α, NF-κB (El-Omar et al., 2000; Polk and Peek, 2010; Wang et al., 2014), and inflammatory mediators facilitate cell proliferation, mutagenesis, oncogene activation, and angiogenesis. IL-1β and TNF-α are proinflammatory acid-suppressive cytokines that are elevated in HP-colonized gastric mucosa (Wroblewski et al., 2010). NF-κB and IL-8 are considered important mediators of gastric pathophysiology in the development of inflammation (Wang et al., 2014). CagA can induce IL-8 expression through NF-κB activation (Polk and Peek, 2010). Activation of NF-κB and up-regulation of IL-8 in gastric epithelial cells are considered important mechanisms of HP-induced carcinogenesis (Brandt et al., 2005).

Furthermore, the effect of HP on GC acts through manipulating host immune systems (Wroblewski et al., 2010). HP and cancer immunomodulatory stromal cells can mediate the immune response to promote tumorigenesis (Navashenaq et al., 2022). HP not only produces immune tolerance by inhibiting T cell function, but also can modify the structure of LPS to evade the recognition of Toll-like receptors (TLRs) pattern recognition receptor family molecules to achieve the purpose of immune escape (Peek et al., 2010). TLRs act in T cell activation, promoting innate immune response and immune tolerance during HP infection (Zhang et al., 2023), which are considered to be the core defects leading to inflammation and cancer development. HP infection can up-regulate the expression of PD-L1 in GC cells by activating the p38 mitogen-activated protein kinase pathway, inhibiting the proliferation of T cells and inducing the differentiation of naive T cells into Treg cells, thereby avoiding immune surveillance and promoting immune escape that ultimately leads to carcinogenesis (Deng et al., 2022).

Other than that, HP infection also disrupts adhesion junctions by inducing the translocation of membrane E-cadherin, β-catenin, and p120 to the cytoplasm of epithelial cells (Wroblewski and Peek, 2013). Strains isolated from patients with lower ferritin levels induce significantly higher levels of IL-8 than strains isolated from patients with the highest ferritin levels, suggesting that iron deficiency in the host increases the virulence of HP and the risk of GC (Noto et al., 2013). HP can inhibit tumor suppressor gene p53 via activating AKT1 to lead to phosphorylation and activation of Human Double Minute 2, which is a potential mechanism for the risk of GC in HP infected individuals (Wei et al., 2010). A study (Tsugawa et al., 2012) provided a molecular link between HP and GC through the specific accumulation of CagA in GC stem-like cells. A recent study (Usui et al., 2023) further demonstrated that HP infection increased the risk of GC associated with germline pathogenic mutations in homologous recombination genes, providing further insight into the gene-environment interaction in the progress of GC.

#### 4.3.3 Emerging new paradigms of HP-induced GC

Notably, some emerging research, such as gut microbiota, immunotherapy, autophagy, exosomes, EMT and GGT may be the focus in the next few years. We discuss the latest hot keywords as follows.

Gut microbiota (GM): GM has been shown to promote the development of HP-associated GC. The lack of commensal microbiota in HP-infected INS-GAS mice can reduce gastritis and delay intraepithelial neoplasia (Lofgren et al., 2011). A vivo study confirmed that limited colonization of gastric flora other than HP can induce the formation of gastric mucosal tumor lesions in INS-GAS mice (Lertpiriyapong et al., 2014). A study found that the changes in GM may be involved in the progression of gastric lesions related to HP infection and provide clues for future evaluation of microbial alterations after HP eradication (Gao et al., 2018). HP may affect the GM through continuous crosstalk with the host immune system. The diversity, composition and function of GM changed after HP infection (Cui et al., 2022). Successful eradication of HP may restore the gastric microbiota to a state similar to that of uninfected individuals and show a beneficial effect on the GM (Guo et al., 2020).

Immunotherapy: Recent studies have shown that HP infection can adversely affect the tumor immune microenvironment and tumor immunotherapy. The overall survival (OS) and progression-free survival (PFS) of HP-positive cancer patients treated with immune checkpoint inhibitors, such as gastric cancer, melanoma and non-small cell cancer patients, were significantly reduced (Che et al., 2022; Oster et al., 2022b; Tonneau et al., 2022), but the specific mechanism is unknown. Some scholars have suggested that HP may reduce the efficacy of immunotherapy by changing the composition of intestinal flora and tumor immune microenvironment, affecting tumor immune response, but there is still a lack of relevant direct evidence (Oster et al., 2022a).

Autophagy: Autophagy is a cell degradation mechanism and may be triggered by HP. Autophagy can mediate ER stress and inflammation in HP-related GC (Mommersteeg et al., 2022). HP-suppressed autophagy promotes the intracellular survival and persistence of pathogens, and also produces an environment conducive to carcinogenesis (Greenfield and Jones, 2013). HP-induced downregulation of p14ARF tumor suppressor gene leads to inhibition of autophagy in infected cells in a p53-independent manner (Horvat et al., 2018). In addition, reactive oxygen species-induced autophagy degradation of HP CagA is specifically inhibited in cancer stem cell-like cells (Tsugawa et al., 2012). HP infection may promote autophagy in human GC cells through Nrf2-mediated heme oxygenase upregulation (Paik et al., 2019).

Exosomes: Exosome is a small extracellular vesicle. Extracellular vehicles (EVs) play an important role in the evolution of malignant tumors because of the genetic material they carry. HP EV is abundant in gastric juice of patients with gastric cancer, which can induce gastric inflammation and may even induce GC, mainly through the selective uptake of gastric epithelial cells to produce inflammatory mediators (Choi et al., 2017). HP infection can induce the up-regulation of activated mesenchymal-epithelial transition factor in exosomes and the tumor-promoting effect on tumor-associated macrophages (Che et al., 2018). Exosomes have been shown to deliver not only various types of genetic information, mainly miRNAs, but also CagA.

Epithelial-Mesenchymal Transition (EMT): EMT is an important part of the invasion, metastasis and multidrug resistance of GC, and it is also one of the key factors for GC. HP CagA promotes EMT in gastric carcinogenesis via triggering oncogenic YAP pathway (Li et al., 2018). The up-regulation of MMP-7 by pathogenic HP is partly dependent on gastrin, and may indirectly increase the level of soluble heparin-binding epidermal growth factor through EMT, which plays a role in the development of GC (Yin et al., 2010). HP infection may trigger TGF-β1-induced EMT pathway and the emergence of GC stem cells, and eradication of HP may prevent the carcinogenesis of GC by inhibiting these two pathways (Choi et al., 2015).

Gamma-glutamyl-transpeptidase (GGT): GGT, an established virulence factor of HP with immunomodulatory properties, can degrade extracellular glutathione, produce reaction products, and increase DNA damage in gastric cells. HP-induced loss of gastric cell survival and viability may be attributed to secreted bacterial GGT activity (Valenzuela et al., 2013). GGT secreted by HP can activate Wnt/β-catenin signaling pathway to promote the occurrence of GC (Meng et al., 2021). A recent study (Baskerville et al., 2023) shows that HP-induced glutathione degradation occurs through an oxidation-independent mechanism driven by the bacterial enzyme GGT, which enhances the ability of bacteria to obtain nutrients from the host.

### 4.4 Limitations of the research

Our study has some potential limitations. Firstly, only the papers indexed in WoSCC database were searched and included, which may not cover all relevant studies from multiple databases worldwide, leading to possible incompleteness of the results. Secondly, current bibliometric tools cannot analyze all contents of papers, resulting in some concrete information being overlooked. High-cited and high-IF paper analysis helped compensate for this disadvantage and limitation. Thirdly, since this study only focused on the current stage of published papers, some newly published papers with significant impact may have been cited less. With the rapid development of research, more papers will become available for analysis.

## 5 Conclusions

Over the last 20 years, interest in HP/GC research has increased. China and Japan were in the leading position and contributed the most to HP/GC research. *Vanderbilt University* and the *US Department of Veterans Affairs* had the maximum Np. The most productive authors were Peek Jr Richard M. and Piazuelo M. Blanca. *Helicobacter* received the most Np, while *Gastroenterology* had the most TC. HP affects the onset and development of GC, as well as the prognosis and effectiveness of treatment of GC. Eradication of HP can not only prevent early GC and metachronous GC but also improve the clinical efficacy of GC treatment. HP may contribute to gastric carcinogenesis through virulence factors, bacterial metabolites, chronic inflammation, and host immunity. Therefore, relevant interventions may represent the next breakthrough in the prevention and treatment of HP-induced GC. Understanding the underlying mechanisms of the links between HP and GC is a fascinating area of research. As HP/GC research advances, gut microbiota, immunotherapy, autophagy, exosomes, EMT, and GGT may emerge as new areas of focus. In summary, this study provides a comprehensive overview of the global status of HP/GC research, enabling scholars to gain a better understand its development trends and identify potential areas for further investigation.

## Data availability statement

The original contributions presented in the study are included in the article/**Supplementary Material.** Further inquiries can be directed to the corresponding authors.

## Author contributions

SY: Conceptualization, Data curation, Formal analysis, Investigation, Software, Visualization, Writing – original draft. SH: Conceptualization, Data curation, Investigation, Software, Visualization, Writing – original draft. HY: Funding acquisition, Methodology, Project administration, Supervision, Validation, Writing – review & editing. XZ: Funding acquisition, Methodology, Project administration, Supervision, Writing – review & editing.
