# Supplementary material for: Cross-talk between Helicobacter pylori and gastric cancer: a scientometric analysis
Source: Front Cell Infect Microbiol. 2024 Jan 31;14:1353094. doi: 10.3389/fcimb.2024.1353094 (PMC10864449; doi:10.3389/fcimb.2024.1353094)
Supplement: Supplementary file 1 [file DataSheet_1.docx]

**Supplementary table.** The papers of historical direct citation network in HP/GC.

| **No.** | **Doi** | **Type** | **Journals** | **First Author** | **Year** | **LCS** | **GCS** |
| --- | --- | --- | --- | --- | --- | --- | --- |
| 1 | 10.1001/jama.291.2.187 | Article | JAMA-J. Am. Med. Assoc. | Wong, BCY | 2004 | 264 | 1064 |
| 2 | 10.1038/nrc1433 | Review | Nat. Rev. Cancer | Hatakeyama, M | 2004 | 103 | 572 |
| 3 | 10.1073/pnas.0504927102 | Article | Proc. Natl. Acad. Sci. U. S. A. | Franco, AT | 2005 | 96 | 381 |
| 4 | 10.1093/jnci/djj393 | Article | JNCI-J. Natl. Cancer Inst. | Kamangar, F | 2006 | 59 | 253 |
| 5 | 10.1053/j.gastro.2007.06.026 | Review | Gastroenterology | Correa, P | 2007 | 66 | 493 |
| 6 | 10.1016/S0140-6736(08)61159-9 | Article | Lancet | Fukase, K | 2008 | 214 | 883 |
| 7 | 10.1073/pnas.0711183105 | Article | Proc. Natl. Acad. Sci. U. S. A. | Ohnishi, N | 2008 | 108 | 421 |
| 8 | 10.7326/0003-4819-151-2-200907210-00009 | Review | Ann. Intern. Med. | Fuccio, L | 2009 | 68 | 285 |
| 9 | 10.1128/CMR.00011-10 | Review | Clin. Microbiol. Rev. | Wroblewski, LE | 2010 | 127 | 896 |
| 10 | 10.1038/nrc2857 | Review | Nat. Rev. Cancer | Polk, DB | 2010 | 93 | 742 |
| 11 | 10.1093/jnci/djs003 | Article | J. Natl. Cancer Inst. | Ma, JL | 2012 | 63 | 314 |
| 12 | 10.1016/j.gie.2011.08.030 | Article | Gastrointest. Endosc. | Maehata, Y | 2012 | 64 | 139 |
| 13 | 10.1136/gutjnl-2012-302240 | Article | Gut | Lee, YC | 2013 | 59 | 240 |
| 14 | 10.1016/j.canlet.2013.08.016 | Review | Cancer Lett. | Wang, F | 2014 | 54 | 458 |
| 15 | 10.1053/j.gastro.2015.09.004 | Review | Gastroenterology | Amieva, M | 2016 | 56 | 501 |
| 16 | 10.1053/j.gastro.2016.01.028 | Review | Gastroenterology | Lee, YC | 2016 | 88 | 490 |
| 17 | 10.1056/NEJMoa1708423 | Article | N. Engl. J. Med. | Choi, IJ | 2018 | 76 | 379 |

**
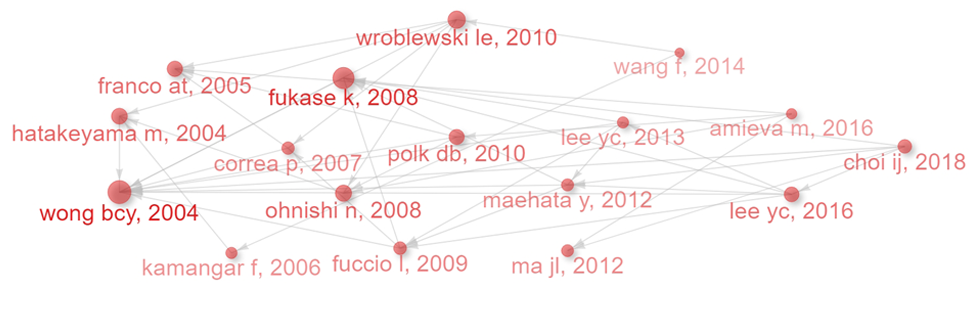
**

**Supplementary figure.** Historical direct citation network in HP/GC research (gray lines represent citation relationships, and each dot represents a paper by author and year).
